# Synergistic improvement of nitrogen and phosphorus removal in constructed wetlands by the addition of solid iron substrates and ferrous irons

**DOI:** 10.1016/j.fmre.2022.10.012

**Published:** 2022-11-02

**Authors:** Liping Tian, Yang Ou, Baixing Yan, Hui Zhu, Huiping Liu, Lei Cheng, Peng Jiao

**Affiliations:** a Key Laboratory of Wetland Ecology and Environment, Northeast Institute of Geography and Agroecology, Chinese Academy of Sciences, Changchun 130102, China; b University of Chinese Academy of Sciences, Beijing 100049, China; c Jilin Provincial Engineering Center of CWs Design in Cold Region & Beautiful Country Construction, Changchun 130102, China; d College of Plant Protection, Jilin Agricultural University, Changchun 130118, China; e College of Resources and Environment, Jilin Agricultural University, Changchun 130118, China

**Keywords:** Sanjiang plain, Ferrous iron, Zero valent iron (ZVI), Substrate, Constructed wetlands (CWs)

## Abstract

Sanjiang Plain is intensively used for rice production, and ditch drainage diffuse pollution prevention is crucial. Groundwater, rich in Fe ions, is the main source of irrigation water in this region. In this study, pyrite and zero-valent iron (ZVI) (sponge iron and iron scraps) were used as substrates to identify the synergistic influence of exogenous Fe^2+^ addition and solid iron substrates on pollutant removal in constructed wetlands. Based on the results, iron substrates hardly improved the ammonia removal, mainly because of the physical structure and oxidation activity. At a hydraulic retention time longer than 8 h, the pollution removal efficiency in the zero-valent iron (ZVI) substrate treatment increased significantly, and the removal of nitrate (NO_3_^−^-N) and total phosphorus (TP) in the iron scrap substrate treatment reached about 60% and 70%, respectively. The high-throughput sequencing results showed a significant increase in the abundance of microorganisms involved in denitrification and phosphate accumulation in biofilms on ZVI substrates. The highest diversities of such microorganisms in biofilms on iron scraps were found for denitrifying bacteria (*Pseudomonas*), nitrate-reducing Fe (II)-oxidizing bacteria (*Acidovorax*), and *Dechloromonas* with autotrophic denitrification and phosphate accumulation, with a 43% cumulative abundance. *Dechloromonas* dominated in the iron sponge substrate treatment. The highest relative abundance of *Acidovorax* was found in the mixed iron substrate (pyrite, sponge iron, and iron scraps) treatment. The addition of ZVI substrate significantly improved the removal of NO_3_^−^-N and TP and reduced the hydraulic retention time through the continuous release of Fe^2+^ and the promotion of microbial growth. When designing constructed wetlands for treating paddy field drainage, the appropriate addition of iron scrap substrates is recommended to enhance the pollutant removal efficiency and shock load resistance of CWs.

## Graphical abstract

**Figure:**
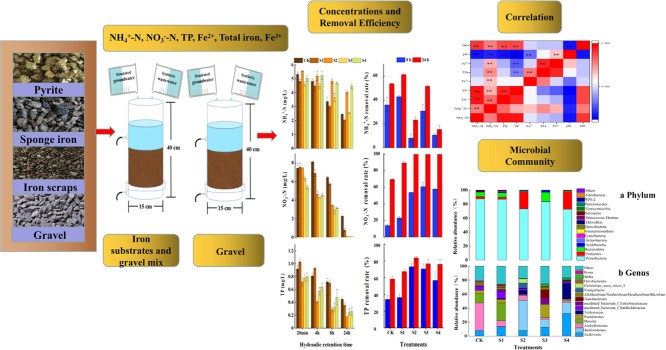

## 1 Introduction

In 2020, the second China Pollution Source Census (2020) showed that 67.2% of phosphorus, 46.5% of nitrogen, 22.4% of ammonia, and 49.8% of the COD in natural aquatic environments were derived from agricultural sources, making the control of agricultural diffuse pollution a key issue [1]. In this context, constructed wetlands (CWs) are important aquatic systems for the reduction of diffuse pollution loads due to their excellent pollutant removal performance, low energy consumption, and simple maintenance [2,3]. They remove pollutants mainly through soil/substrate adsorption, plant uptake, root oxygen secretion, and microbial degradation. However, the spatial and temporal variability of purification efficiency of wetlands is influenced by various factors such as climate, plant type and growth, and the length of the hydraulic residence time [3].

In constructed wetlands, electron donors are a major constraint to denitrification. Some microorganisms can use Fe^2+^ as an electron donor to convert NO_3_^−^-N to N_2_ via denitrification when the electron donors, such as organic carbon sources, are limited [4]. The iron-nitrogen coupling process involves the inter conversion of Fe^2+^ and Fe^3+^ and the coupling with nitrification and denitrification processes [5]. There are two mechanisms by which Fe^2+^ is involved in denitrification: first, Fe^2+^ can react with NO_3_^−^-N, and second, Fe^2+^ takes part in the biological denitrification with NO_3_^−^-N. In the presence of iron, NO_3_^−^-N relies on the reductive metabolism of certain microorganisms that can reduce NO_3_^−^-N while oxidizing Fe^2+^. In addition, due to solubility limitations, Fe^2+^ and Fe^3+^ bind toPO_4_^3−^ in the water and sink, thereby directly removing NO_3_^−^-N from the water. On the other hand, Fe ions undergo hydrolysis and various polymerisation reactions through dissolution and water uptake to form linearly structured polynuclear light group complexes [6]. These ‘advanced’ polymers have an indirect effect on phosphate removal from wastewater through adsorption and complexation.

The use of iron to remove nitrogen and phosphorus from wastewater has been applied in large-scale wastewater treatment plants and large constructed wetlands, obtaining promising results [7], [8], [9]. However, there are few reports on the application of this technique in micro-wetlands converted from degraded wetlands or artificial drainage ditches. As agricultural diffuse pollution is scattered, hidden, random, uncertain, widespread, and not easily monitored, centralized treatment is challenging, and the use of micro-wetlands for pollution removal, with the addition of small amounts of exogenous iron, has become an important research field.

Sanjiang Plain in Heilongjiang Province, China, is an important commercial grain base and a large-scale intensive paddy field distribution area. With the significant increase in grain production in recent years, the risk of surface pollution from ditch drainage has increased rapidly. Most of the rivers and lakes in the region are boundary rivers and lakes, which are highly sensitive and prone to international disputes over water pollution; they are also key areas for agricultural diffuse pollution prevention and control, requiring urgent national strategies. The groundwater in Sanjiang Plain, which is used for agricultural irrigation, is generally rich in Fe, with a higher Fe^2+^content compared to Fe^3+^; the soluble Fe (mainly Fe^2+^) content varies from 0.03 to 21.00 mg/L, with an average of 5.48 mg/L [10]. Because of the large number of depressions, abandoned large ditches, and degraded wetlands on the Sanjiang Plain, there is ample space for the construction of micro-wetlands. Such wetlands could effectively treat agricultural drainage high in N, P, and Fe^2+^, thereby controlling diffuse pollution [11]. However, the spatial variability of Fe^2+^ concentrations in groundwater makes it difficult to improve wetland nitrogen and phosphorus removal efficiencies using only natural sources of Fe^2+^. Many studies have found that ZVI as an iron substrate can not only participate in denitrification directly as an electron donor, but also, the Fe^2+^ generated after the dissolution of ZVI provides electron donors for denitrification [12], [13], [14]. In this context, much attention has been paid to the use of pyrite, sponge iron, and iron scraps as ZVI substrates for improving nitrogen and phosphorus removal efficiencies in wetlands [8,15,16]. However, studies on their synergy with low concentrations of Fe^2+^ to improve nitrogen and phosphorus reduction efficiencies are scarce. In particular, research on the selection and combination of zero-degree iron substrate types in the design of constructed wetlands for the treatment of unstable pollution sources, such as paddy field drainage, needs to be strengthened. Therefore, the objectives of this study were to (1) evaluate the synergistic effects of different iron substrates and Fe^2+^ on CW performance; (2) identify the bacterial community structures in the biofilms of different substrates; (3) determine the optimal CW operation and design parameters.

## 2 Materials and methods

### 2.1 Substrates

Pyrite, sponge iron, and iron scraps were purchased from Zhengzhou, Henan Province, China, at particles sizes of 1–2 mm. Gravel was obtained from Changchun, Jilin Province, China, at a diameter of 1–2 cm, a specific surface area of 0.03 g/m^2^, and a pore size of 82.99 nm.

### 2.2 Experimental setup

**Fig. 1:**
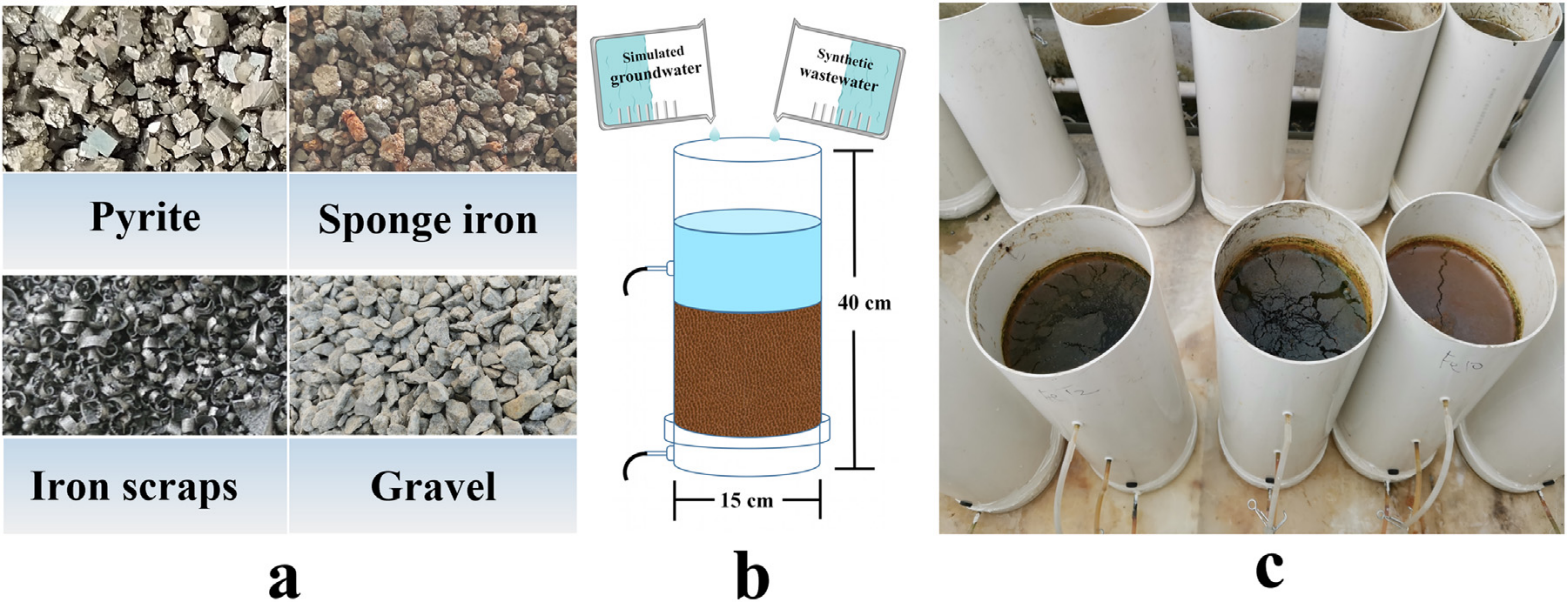
**Substrates (a), schematic diagram (b), and photo of the CWs microcosms (c)**.

The micro-scale CWs without plants were composed of 15 PVC cylinders with a thickness of 0.2 cm, a height of 40 cm, and a radius of 15 cm; they had a sealed bottom and an unsealed top (Fig. 1). The outlet was set at 15 cm from the bottom, and the cylinders were placed in a greenhouse in the Northeast Institute of Geography and Agroecology, Chinese Academy of Sciences, at a temperature of 25–30 °C. The following five treatments were established: CK, gravel substrate; S1, substrate of pyrite and gravel (1:5 v/v); S2, substrate of sponge iron and gravel (1:5 v/v); S3, substrate of iron scraps and gravel (1:5 v/v); S4, substrate of pyrite, sponge iron, iron scraps, and gravel (1:1:1:3 v/v). The volume ratios were set for two main reasons: 1) cost, as the main reason for the widespread use of CWs is their low cost, minimizing the amount of iron used is an effective means of reducing construction costs; 2) drainage, as blockage is the most common problem in the operation of CWs. The larger the proportion of gravel, the higher the porosity of the wetland substrate facilitated drainage. Therefore, based on the above reasons and the available information on constructed wetland design, the volume ratio of iron substrates to gravel in this study was 1:5. The substrates were washed with tap water before being placed into the CWs. Each treatment was performed in three replicates, with a total of 15 reactors.

### 2.3 Operation and sampling

To avoid interferences from other factors, the wastewater in the CWs was configured manually. The concentration of the Fe^2+^ solution was derived from our previous study [11]. Table 1 shows the concentrations and compositions of the simulated wastewater and the Fe^2+^solutions. The experiment was divided into two periods, namely the stable period (approximately 30 days) and the operation period (10 days). The CWs were particularly designed for the treatment of paddy ditch drainage; based on a field survey, large-scale drainage of paddy fields on the Sanjiang Plain only occurs two or three times a year, lasting for about 1 week. Therefore, the operation period was set to 10 days, mainly to examine whether the pollutant removal efficiency of CWs could meet the demands within this relatively short period.

**Table 1:** **The composition and concentration of the synthetic wastewater and simulated groundwater (mg/L)**.

	Synthetic wastewater			Simulated groundwater
Nutrients	NH_4_^+^-N	NO_3_^−^-N	TP	Fe^2+^
Composition	NH_4_Cl	KNO_3_	K_2_HPO_4_	FeSO_4_•7H_2_O
Concentration	10	14	2	10.00

During the stable period, 1/4 Hoagland nutrient solution was fed to the micro-scale CWs to maintain biofilm activity and moisture conditions. After the stable period, the effects of iron substrates and hydraulic retention times (HRTs) on pollutant removal were determined. All experiments were run for 10 cycles, with an HRT of 24 h throughout the experiments. The volume ratio of simulated wastewater to Fe^2+^ solution was 1:1, and 2 L of simulated wastewater was added to the reactor in advance, followed by the addition of Fe^2+^solution so that the final Fe^2+^ in the CWs was 5 mg/L. Samples were taken at 20 min, 4 h, 8 h, and 24 h after the start of the experiment, filtered through a 0.45-μm membrane, and stored at 4 °C until analysis. The levels of NO_3_^−^-N, NH_4_^+^-N, TP, Fe^2+^, Fe^3+^, and total iron (TFe) were determined using an automated chemical analyzer (Mode Smart Chem 200, Rome, Italy). The pH and dissolved oxygen (DO) values were analyzed immediately using a portable water quality analyzer (HQ40d, HACH, Loveland City, Colorado, USA).

### 2.4 Microbial analysis

Substrate samples were sent to Sangon Biotech Co., Ltd. (China), and DNA was extracted using a microbial DNA extraction kit (Biocolors, Shanghai). The V3–V4 regions of 16SrRNA genes were amplified using primers 338F (5-ACTCCTACGGGAGGCAGCAG-3′) and 806R (5-GGACTACHVGGGTWTCTAAT-3′). To identify the microbial community, Illumina high-throughput sequencing was conducted to analyze the microbial diversity and structure of the samples of each treatment. For this, the database RDP (Ribosomal Database Project) was used (http://rdp.cme.msu.edu/index.jsp).

### 2.5 Statistical analysis

All data were analyzed using the software package SPSS 19.0 (SPSS Inc., Chicago, IL, USA). The results are presented as means ± standard deviation (SD). Means of the different treatments were compared by one-way analysis of variance (ANOVA) with Tukey´s HSD test at a significance level of 0.05. All figures were designed and plotted using Origin 2021 (OriginLab Inc., Northampton, MA, USA).

## 3 Results

### 3.1 Overall performance of each treatment

#### 3.1.1 Ammonia removal

**Fig. 2:**
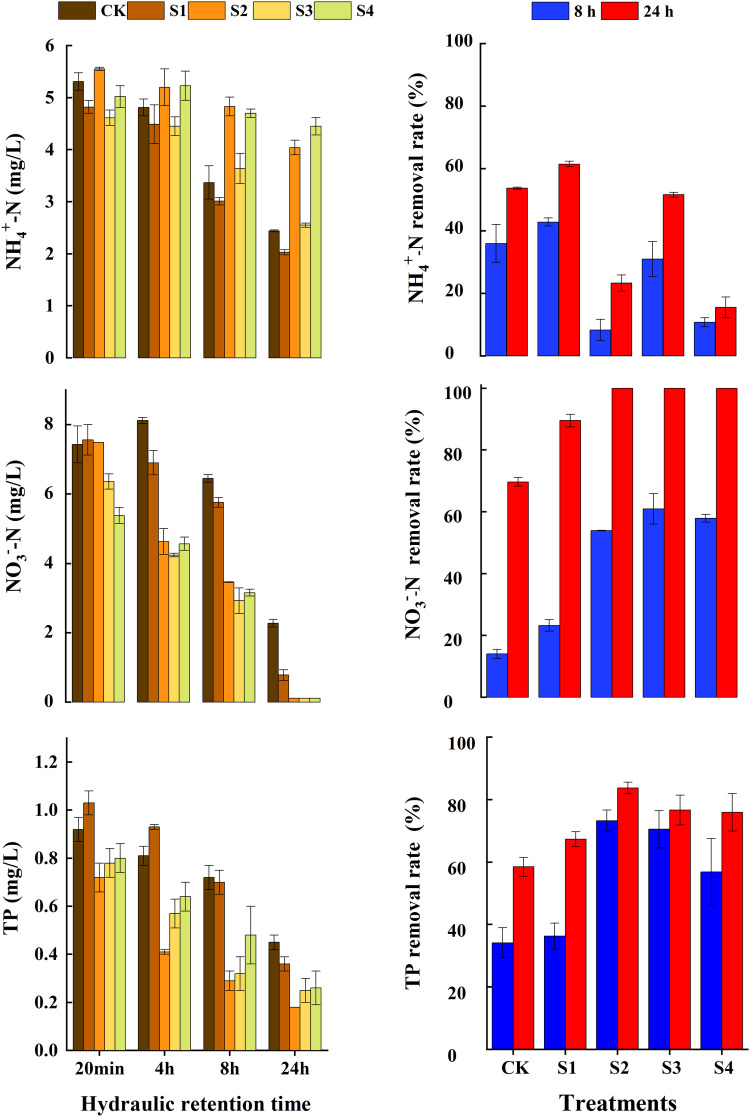
**NH**_**4**_^**+**^**-N, NO**_**3**_^**−**^**-N and TP concentrations and removal rate at HRT-8 h and 24 h in CWs.** Note: CK, S1- pyrite, S2 - sponge iron, S3 - iron scraps and S4 - pyrite, sponge iron and iron scraps.

With the increase in the HRT, the NH_4_^+^-N concentration in the different CWs showed a decreasing trend, and the effluent concentrations of some treatments were significantly lower than the influent concentrations (Fig. 2). After an HRT of 4 h, the removal rates of S1 and S3 were significantly (*p* < 0.05) higher than those of S4 and S2. The 8-h removal rates of S1, CK, and S3 were 42.89%, 36.01% and 31.01%, respectively, while those of S4 and S2 were 10.82% and 8.29%, respectively. The average removal rates in 24 h for all treatments were 15.57%–61.46%. During the operation period, the treatments using pyrite (S1) and iron scraps (S3) showed higher NH_4_^+^-N removal rates of 61.46% and 51.61%, respectively, while the other two iron substrate treatments (S2 and S4) only showed reduction rates of around 20%.

#### 3.1.2 Nitrate removal

The NO_3_^−^-N concentration in the solid iron substrate treatments decreased sharply as the HRT was prolonged, whereas its concentration remained largely stable in CK (Fig. 2). At an HRT of 20 min, the NO_3_^−^-N removal rate increased by 14.20% and 27.22% for S3 and S4, respectively, compared to CK. When the HRT exceeded 4 h, the NO_3_^−^-N removal rate of ZVI substrate treatments was significantly higher than that of CK (*p* < 0.05), and at an HRT of 8 h, the removal rate of the iron scrap treatment had reached 61.01%; in contrast, the pyrite substrate treatment only showed a removal rate of 23.28%. At an HRT of 24 h, except for CK, the NO_3_^−^-N removal rates for all treatments were greater than 89.56%, and those of the ZVI substrate treatments were close to 100%.

#### 3.1.3 Total phosphorus removal

The effluent TP concentrations of the ZVI treatments were significantly (*p* < 0.05) lower than the influent concentrations (Fig. 2). During the entire operational period, the highest TP removal rate occurred in the sponge iron treatment (S2), with 34.62% at an HRT of 20 min, while a removal rate of only 14.87% was observed in CK. At an HRT of 4 h, the removal rate of S2 reached 62.07%, which was higher than the 35.75% of CK. At an HRT of 8 h, S2 showed a removal rate of 72.93%, which was higher than the 38.91% of CK. At an HRT of 24 h, the TP removal rates of all treatments were close to 60% and above.

### 3.2 Variations in the physiochemical factors in CWs

#### 3.2.1 Effluent iron concentration

**Fig. 3:**
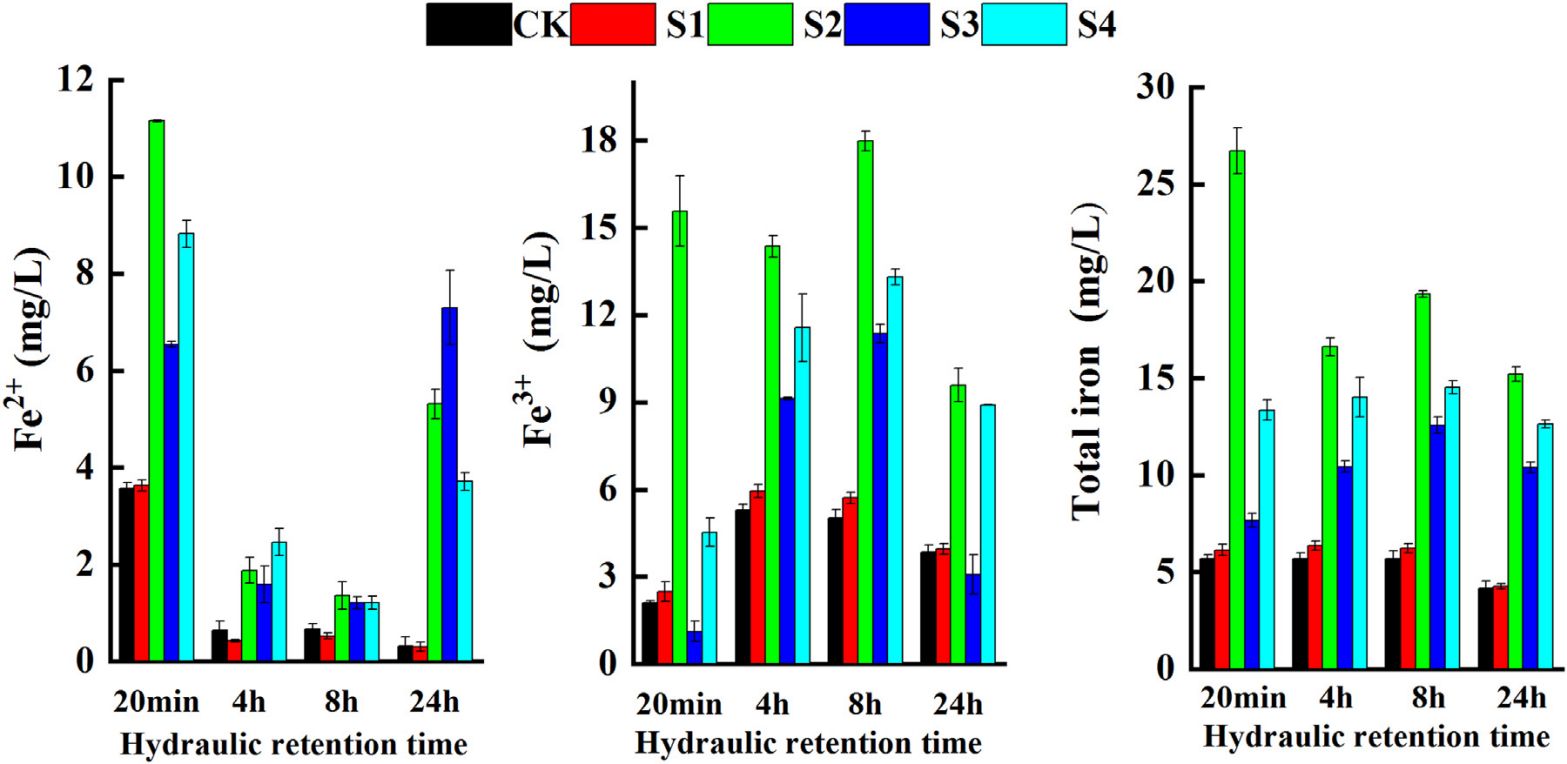
**Variations of Fe**^**2+**^**, Fe**^**3+**^**and total iron concentrations in CWs**.

As shown in Fig. 3, the effluent Fe^2+^ concentrations in all treatments showed a trend of first decreasing and then increasing. At an HRT of 4 h, Fe^2+^ showed a decreasing trend. At HRTs between 8 and 24 h, Fe^2+^ in the ZVI treatments showed an increasing trend, while the Fe^2+^concentrations in S1 and CK showed a decreasing trend. At an HRT of 24 h, the concentrations of Fe^2+^in the ZVI treatments were 3.71 mg/L (S4) to 7.30 mg/L (S3), while in the CK and pyrite treatment, the levels were only around 0.30 mg/L. The addition of ZVI significantly increased the Fe^2+^ level in the CWs, which was not observed for pyrite addition. Except for S2, the Fe^3+^ concentrations in all five treatments showed the opposite patterns when compared to Fe^2+^. At an HRT of 8 h, higher concentrations of Fe^3+^ were observed in S2, S3, and S4, at levels of 17.98, 11.37, and 13.30 mg/L, respectively. During the entire operation period, although the total iron (TFe) concentration largely differed among the five treatments, the change trend with HRT was similar. At an HRT of 24 h, the concentrations of TFe in the ZVI treatments ranged from 10.39 mg/L (S3) to 15.20 mg/L (S2), while its concentration in CK and the pyrite treatment was only about 4 mg/L.

#### 3.2.2 Effluent pH and DO

**Fig. 4:**
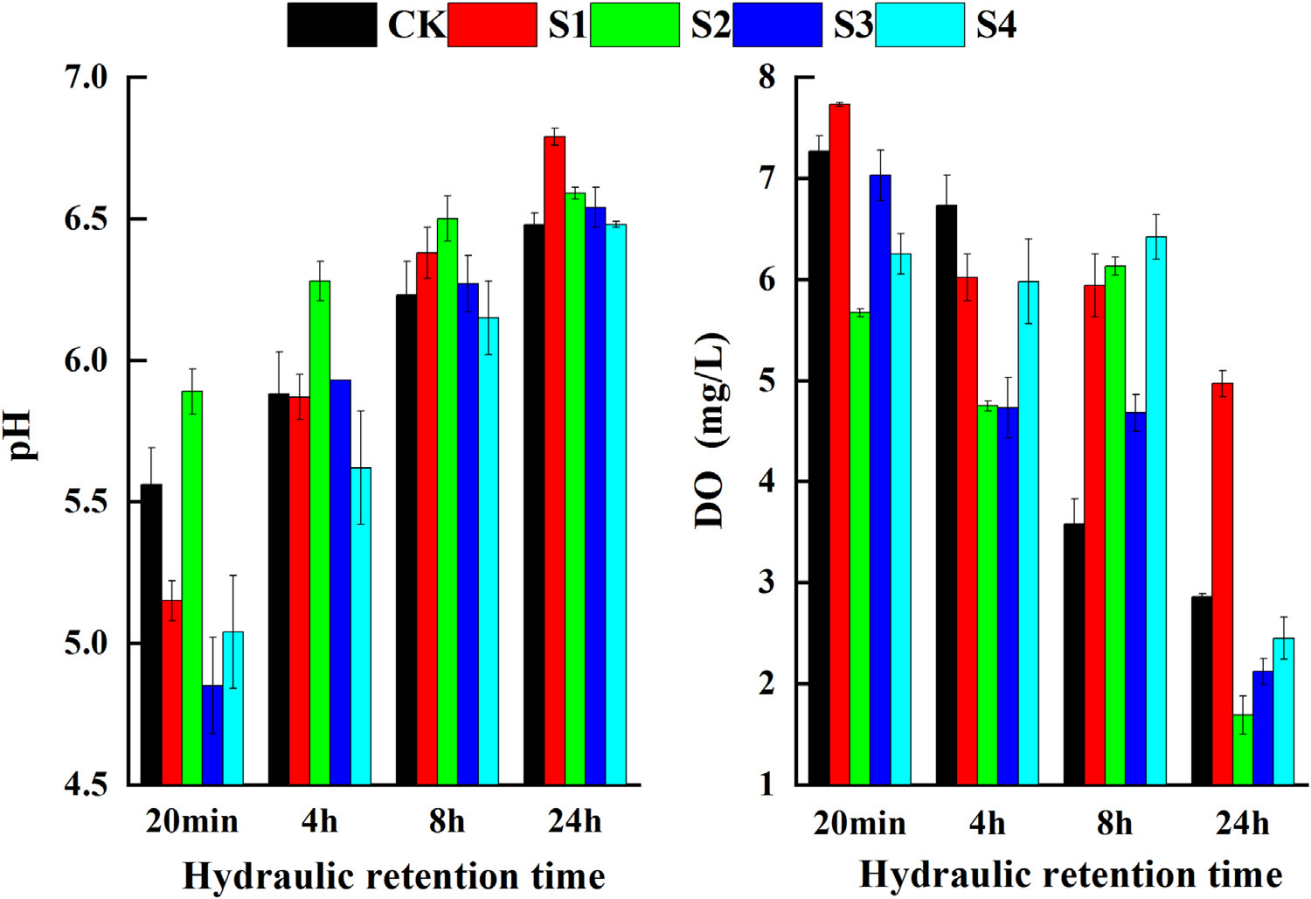
**Variations of DO and pH in CWs**.

As shown in Fig. 4, throughout the experiment, the effluent pH increased gradually with an increasing HRT. For a 20-min HRT, there was significant difference in pH in all five treatments (*p* < 0.05). At an HRT of 24 h, the pH of all treatments had reached their highest levels, without any significant differences among the treatments. Contrary to pH, the DO levels of all treatments fluctuated and decreased with an increasing HRT. At an HRT of 20 min, the DO concentration ranged from 5.67 mg/L (S2) to 7.73 mg/L (S1), whereas at an HRT of 24 h, the DO concentration ranged from 1.69 mg/L(S2) to 4.97 mg/L(S1). There was a significant difference in the DO levels among the five treatments.

### 3.3 Microbial communities in CWs

#### 3.3.1 Microbial diversity

A comparison of the alpha-diversity estimators indicated that high-throughput sequencing generated reliable data for biodiversity analysis (Table 2). The Shannon index refers to the comprehensive assessment for the richness and evenness of microbial communities [13], whereas the Simpson index reflects the microbial diversity in the samples. Larger Simpson index values imply a lower diversity of the community. In this study, except for the pyrite treatment, the Shannon index was significantly lower in the treatments than in CK (*p* < 0.05). This indicates that the addition of ZVI substrates significantly reduced microbial diversity and evenness in the CWs and that the microbial diversity decreased further with increasing iron concentrations. The biodiversity estimators Chao1 and Ace were used to evaluate community richness [17], both estimators showed lower levels in the treatments with iron substrates than in the CK, suggesting that solid iron reduces the microbial richness. This finding is in agreement with a previous study in which sponge iron (s-Fe0) was negatively related with microbial richness in CWs [13].

**Table 2:** **The characteristics of microbial community diversity in CWs**.

Systems	Feature	Shannon	Chao1	Simpson	ACE	Coverage
S1	298.50 ± 0.50^a^	4.86 ± 0.17^a^	333.87 ± 2.71^a^	0.91 ± 0.02^a^	324.95 ± 3.22^a^	1.00 ± 0.0001^a^
S2	242.50 ± 2.50b^c^	3.39 ± 0.12^b^	294.94 ± 5.31^b^	0.78 ± 0.01^b^	274.50 ± 0.49^b^	1.00 ± 0.0001^b^
S3	203.50 ± 9.50^b^	4.46 ± 0.15^c^	246.34 ± 2.14^b^	0.92 ± 0.01^a^	245.58 ± 2.78^b^	1.00 ± 0.0001^b^
S4	238.50 ± 2.50^c^	3.42 ± 0.30^b^	277.84 ± 1.00^b^	0.80 ± 0.04^b^	276.74 ± 1.84^b^	1.00 ± 0.0001^b^
CK	332.00 ± 1.00^a^	4.49 ± 0.10^c^	351.87 ± 2.21^a^	0.85 ± 0.03^c^	345.49 ± 0.93^a^	1.00 ± 0.0001^a^

Note: CK, S1- pyrite, S2 - sponge iron, S3 - iron scraps and S4 - pyrite, sponge iron and iron scraps.

#### 3.3.2 Microbial composition

**Fig. 5:**
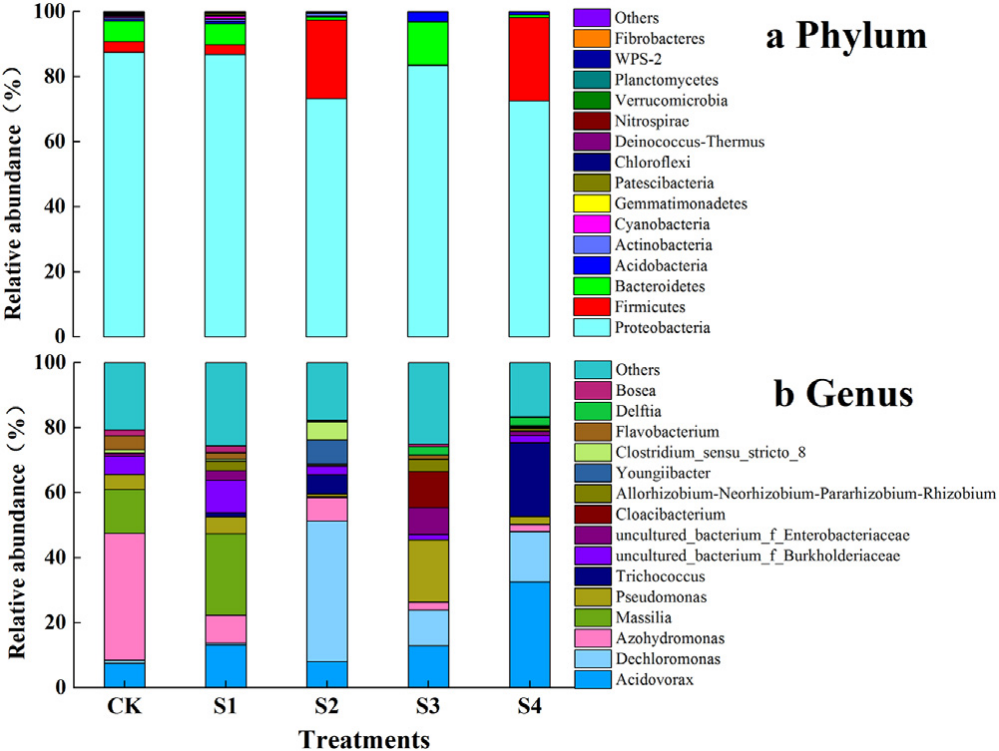
**Relative abundance of microbial communities in CWs**.

Based on high-quality sequencing of the 16S rRNA gene, the microbial community structures of the five treatments were analyzed at phylum and genus level. Taxa with relative abundances less than 1% and/or unclassified taxa were defined as “Others”. Fig. 5a shows that Proteobacteria was the dominant phylum in all treatments, with relative abundances ranging from 72.56% (S4) to 86.85% (CK), although the values did not differ significantly among the treatments. Firmicutes was the second most abundant phylum, with relative abundances ranging from 0.35% (S3) to 25.59% (S4), followed by Bacteroidetes, with abundance levels from 0.86% (S4) to 13.20% (S3). The relative abundances of Firmicutes in the treatments with sponge iron (S2 and S4) were significantly higher than those in the other treatments (*p* < 0.05), whereas in S3, the relative abundance of Firmicutes was significantly lower.

As shown in Fig. 5b, the distribution of bacterial communities at genus level varied widely among the five treatments. *Acidovorax* (7.54%–32.65%), *Dechloromonas* (0.47%–43.23%), and *Azohydromonas* (2.06%–39.04%) were the dominant genera in the five treatments. The relative abundance of *Acidovorax* in iron substrates was higher than that in CK. The highest relative abundance of *Dechloromonas* was observed in S2 and significantly higher than those in the other treatments. However, the lowest relative abundances of *Dechloromonas* were found for S1 and CK. The relative abundance of *Azohydromonas* in CK was significantly higher than that in the other treatments (*p* < 0.05). It should be noted that the relative abundances of *Pesudomonas* and *Trichococcus* in iron substrate treatments were higher than those in CK. The genus *Massilia* showed the highest relative abundance in S1, with 25.12%.

## 4 Discussion

### 4.1 Influence of iron on N and P removal in CWs

As shown in Fig. 3, in CK, the Fe^2+^ effluent concentration was close to 0 at an HRT of 4 h, whereas the Fe^3+^ concentration was close to 5 mg/L, indicating that Fe^2+^ from groundwater irrigation was rapidly depleted in the early stages of paddy drainage treatment. The iron substrates, on the other hand, accelerated the release of Fe^2+^, and after 24 h, the Fe^2+^ in the sponge iron and iron scraps substrate treatments had reached half of the initial concentration (Fig. 3). Furthermore, as seen in Fig. 5, the microorganisms involved in denitrification and phosphate accumulation in the substrate biofilm were five times more abundant in the iron sponge treatment than in the CK. This leads us to infer that the addition of ZVI substrates to CWs with low Fe^2+^concentrations accelerates synergistic biological and chemical reactions, which not only effectively reduces the peak concentrations of nitrogen and phosphorus in the early stages of paddy drainage treatment but also sustains the reduction efficiency of the CW. These findings therefore provide further evidence that the synergy of these two enhancements can facilitate the improvement of the performance of constructed wetlands.

There are two main pathways for iron substrates to enhance the nitrogen and phosphorus removal abilities of wetlands. One is to enhance the oxidation or complexation precipitation of pollutants by changing the physical-chemical conditions of the wetlands, and the other is to promote the growth of a certain microbial population in a targeted manner to improve the biochemical reaction rate and enhance the use of pollutants for microbial growth [18]. In this study, the ammonia removal efficiencies of the sponge iron (S2) and mixed with sponge iron (S4) treatments were lower compared to those of the other treatments. This can largely be explained by the loose and porous internal structure of sponge iron, providing a 5–10 higher specific surface area than iron scraps, consequently allowing the rapid and complete oxidation of oxygen and iron in water and a significant reduction in the ammonia nitrogen oxidation capacity of wetlands [19]. In addition, the pyrite substrate treatment not only showed lower nitrate and total phosphorus reduction rates but also lower relative abundances of nitrate-reducing or phosphate-accumulating microbial communities (*Acidovorax, Dechloromonas*, and *Pesudomonas*), indicating that the iron substrate type is closely related to the pollutant removal rates [20,21].

**Fig. 6:**
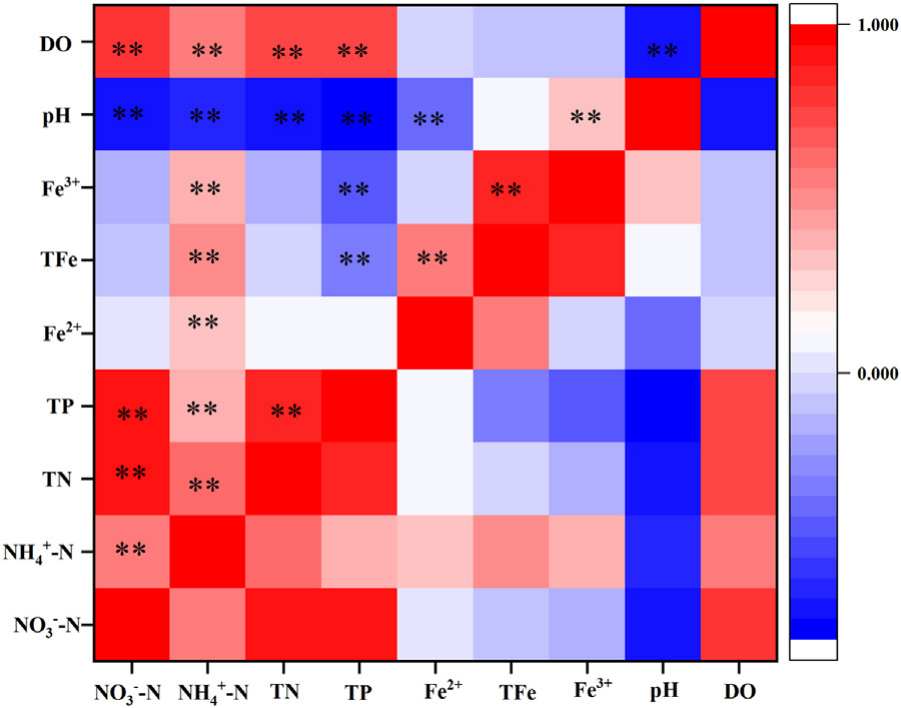
**Correlation between nitrogen, phosphorus, iron and physicochemical parameters in CWs.** Note: * indicates *P* < 0.05, ** indicates *P* < 0.01.

The addition of iron substrates significantly enhanced the NO_3_^−^-N removal rates in the CWs (Fig. 2). The concentration of NO_3_^−^-N was positively correlated with DO (*p* < 0.01) and negatively with pH (*p* < 0.01) (Fig. 6). Under anoxic or anaerobic environments, NO_3_^−^-N could be directly reduced by ZVI, simultaneously promoting the corrosion of ZVI to Fe^2+^ and, consequently, providing electron donors for denitrification. Some studies have suggested that Fe^2+^ in ZVI systems can affect iron corrosion by DO, oxygen production, and the reduction of NO_3_^−^-N [18]. Neither NO_3_^−^-N nor DO could be effectively removed by ZVI without an increase in Fe^2+^ in the CWs. In the presence of Fe^2+^, NO_3_^−^-N and DO could be removed simultaneously, without interactions [22]. This was consistent with the results of this study that the NO_3_^−^-N and DO concentrations decreased in CWs containing ZVI substrates when the Fe^2+^concentration was maintained above 2 mg/L. These micro-batteries use high-potential carbon as the cathode and low-potential iron as the anode in an acidic electrolyte solution where an electrochemical reaction occurs to produce Fe^2+^. Several studies have found that Fe^2+^ significantly reduces the concentration of NO_3_^−^-N in CWs, suggesting that Fe^2+^ promotes denitrification [11,23]. In addition, both substances have a considerable surface area, which favors the attachment of denitrifying microorganisms [24,25].

The TP removal by CWs mainly occurs via substrate capture, plant uptake, and microbial use, where substrate adsorption and precipitation formed by different ions released from the substrate are essential to achieve efficient TP removal. In this study, TP was significantly positively related with DO and significant negatively related with Fe^2+^, Fe^3+^, and pH (Fig. 6). In the iron substrate treatments, Fe^2+^ and Fe^3+^ precipitate with PO_4_^3−^ because of limited solubility [26], resulting in the direct removal of phosphate. Furthermore, via various polymerization reactions, more than 10 different types of polynuclear light complexes with linear structure are generated [27]. These "advanced" polymers indirectly remove phosphate from wastewater through adsorption and complexation. In this sense, the Fe^3+^ concentration is the key to TP removal in constructed wetlands with different iron substrates. The highest removal rate of TP in the pyrite treatment (S2) may be explained as follows: this treatment showed the maximum Fe^3+^concentration, and compared to pyrite and iron scraps, sponge iron has a porous surface that could adsorb more phosphates. This is supported by previous studies [13]. As shown by the microbial community analysis, the relative abundance of *Dechloromonas* in S2 was higher than those of the other genera. *Dechloromonas* is a typical denitrifying bacterium that not only participates in autotrophic denitrification but can also accumulate phosphate [28], which resulted in the highest TP removal in S2.

### 4.2 Impact of environmental factors on the performance of CWs

Various studies have shown that pH and DO play key roles in the removal of nitrogen and phosphorus pollutants in constructed wetlands, and changes in these two parameters not only indirectly reflect the characteristics of wetland substrates or design but also the removal of nitrogen and phosphorus. For example, in constructed wetlands with pyrite as substrate, pyrite acted as an oxidizing agent [29], facilitating a chemical oxidation reaction between iron sulfide (FeS_2_) and dissolved oxygen [12], simultaneously stimulating microbial growth and depleting dissolved oxygen in the influent, thereby enabling the formation of an anaerobic environment and facilitating denitrification [30]. In other studies, poorly crystallized iron hydroxide [31], Fe^2+^, and Fe^3+^ generated from pyrite at the bottom of constructed wetlands during heavy oxygen consumption could improve phosphorus removal efficiency by adsorption or precipitation of PO_4_^3−^[15,24]. In another study, iron sponge substrate both removed large amounts of oxygen from the effluent, ensuring an anaerobic environment for biological denitrification in the wetland [32], and effectively produced [H] and/or H_2_ as well as Fe^2+^ through micro-electrolysis, providing the H^+^or neutralizing released OH^−^ ions required in chemical denitrification. In our study, pH was an important parameter influencing the kinetic process of denitrification, and at alkaline pH values, the transfer of electrons from sponge iron to NO_3_^−^-N was inhibited. Si et al. [12] suggested a significant negative correlation between influent NO_3_^−^-N concentration and effluent pH in artificial wetlands with sponge iron substrate because of the increase rate of chemical denitrification. Nguyen et al. [33] noted that at lower pH values, the OH^−^ produced during ZVI corrosion was heavily depleted, favoring the chemical reduction of NO_3_^−^-N to NH_4_^+^-N, whereas a higher pH inhibited biological denitrification. An adequate pH is the key to achieving efficient pollutant removal in wetlands with ZVI substrates [34] as the NO_3_^−^-N removal efficiency is significantly affected by the initial pH of the solution. In a previous study, the initial value was positively correlated with the removal of NO_3_^−^-N when the pH was greater than 3.0, whereas nitrite removal was more efficient at a pH of about 7.0 [35]. In this study, the initial pH was 4.5–7.0, and therefore, the iron substrates significantly improved the NO_3_^−^-N removal in the CWs. The main reason for higher NO_3_^−^-N removal in S2 and S3 was not only due to the relatively low DO level, but also related to the higher Fe^2+^ concentration (Fig. 6), as sponge iron and iron scraps immersed in the wastewater could form micro primary batteries [36].

### 4.3 Role of iron in bacterial community structures shifts

According to a previous study, ZVI can cause oxidative damage to microorganisms, subsequently leading to cell death [37]. This can be explained by the fact that the introduction of sponge iron and iron scrap particles into CWs causes changes in the microbial community composition and negatively affects microbial richness and uniformity. In addition, during ion release, DO and protons in the wastewater will be absorbed and consumed, thereby changing the pH and DO levels and, at the same time, altering the environmental conditions for microbial growth, consequently affecting the structure of the microbial community. Proteobacteria and Firmicutes showed high abundance at phylum level in all the treatments (Fig. 5a) and they were not only dominant phyla in CWs but also Fe(II) oxidizing microorganisms. Proteobacteria play an important role in organic matter degradation, nitrification, denitrification, and phosphorus removal [38]. In addition, Bacteroidetes, also highly abundant, were associated with denitrification and detected in all treatments. This might explain the continuous decrease in nitrogen and phosphorus concentrations in CK.

The genus *Acidovorax*, belonging to nitrate-reducing Fe(II) oxidizing strains, may promote Fe(II) oxidation and nitrate reduction through direct enzymatic pathways and indirect GR-mediated processes [28]. In this study, the relative abundance of *Acidovorax* in iron substrate treatments was higher than that in CK, most likely because of the higher removal of NO_3_^−^-N and TP in the treatments with iron substrates. *Dechloromonas* is a typical genus of denitrifying bacteria, performing with autotrophic denitrification and phosphate accumulation [28], and is an important heterotrophic denitrifying bacterium [8]. It is characterized by the anaerobic oxidation of chelated Fe(II) combined with nitrate reduction at neutral pH and is known as a perchlorate- and nitrate-reducing agent [39]. The relative abundance of *Dechloromonas* was highest in S2, which explains the higher combined removal of nitrogen and phosphorus in S2 compared to the other treatments. It was found that relative abundance of *Azohydromonas* in CK was significantly higher than that in the other treatments (*p* < 0.05). *Azohydromonas* is a nitrogen-fixing microorganism that plays an important role in the nitrogen cycle [40], which explains the lowest denitrification rate in CK.

Some studies have indicated that adding iron substrates to leaching iron ions is beneficial to enrich bacteria by enhancing microbial aggregation and reducing bacterial leaching [41]. The relative abundance of *Acidovorax* in S4 was significantly higher than that in the other treatments, indicating that the combination of pyrite, sponge iron, and iron scraps was beneficial for the enrichment of *Acidovorax*. Similarly, the relative abundance of *Dechloromonas* in S2 was significantly higher than that in the other treatments (*p* < 0.05), suggesting that sponge iron facilitates the growth of *Dechloromonas. Trichococcus* is an Fe(III)-reducing or electroactive iron-reducing bacterium [42] that reduces oxidized Fe^3+^ to Fe^2+^ and continues to supply electrons to nitrate, potentially participating in nitrogen biodegradation [43]. *Pseudomonas* not only plays an important role in heterotrophic denitrification but also participates in NH_4_^+^-N oxidation and TP removal processes, making it an important genus for the removal of nitrogen and phosphorus from water [15,44]. In this study, the relative abundance of *Pseudomonas* in the iron scrap treatment was significantly higher than that in the other treatments [Fig. 2], with the highest pollutant removal except for NO_3_^−^-N [Fig. 5]. These findings suggest that iron scraps are an optimal substrate for paddy drainage treatment in CWs. Some species belonging to the genus *Massilia* can dissolve phosphate [12,45] and participate in the nitrogen and iron cycles in soil [46,47]. Their presence at high abundances may therefore be one of the reasons for the decrease in nitrogen and phosphorus concentrations in S1 and CK.

## 5 Conclusion

In this study, the effects of solid substrates (pyrite, sponge iron, and iron scraps) on pollution removal efficiency and the characteristics of the microbial communities in the attached biofilms were assessed using simulated CWs and high-throughput sequencing with exogenous Fe^2+^ addition. The ZVI substrate had the greatest impact on the physicochemical properties of the wetland, resulting in a sustained release of Fe^2+^ and reduced dissolved oxygen levels, facilitating the formation of an anaerobic environment that accelerated denitrification. When the hydraulic retention time exceeded 8 h, the removal of NO_3_^−^-N and TP in the iron scrap substrate treatment had reached about 60% and 70%, respectively, an increase by 40% compared to the control. The ZVI substrates also promoted the development of microorganisms that could denitrify and accumulate phosphorus. For example, the addition of iron scraps significantly increased the diversity microorganisms such as denitrifying bacteria (*Pesudomonas*), nitrate-reducing Fe (II)-oxidizing bacteria (*Acidovorax*), and *Dechloromonas* with autotrophic denitrification and phosphate accumulation capacity, with a cumulative abundance of 43%. In summary, the synergistic effect of exogenous Fe^2+^ and ZVI substrates not only effectively reduced the peak concentrations of nitrogen and phosphorus pollutants at the beginning of paddy field drainage treatment but also considerably shortened the HRT. These findings provide information about the important operational and design parameters for constructed wetlands to mitigate diffuse pollution from paddy field drainage in Northeast China.

## Declaration of competing interest

The authors declare that they have no conflicts of interest in this work.
